# Changes in cell walls lignification, feruloylation and p-coumaroylation throughout maize internode development

**DOI:** 10.1371/journal.pone.0219923

**Published:** 2019-07-30

**Authors:** Yu Zhang, David Legland, Fadi El Hage, Marie-Françoise Devaux, Fabienne Guillon, Matthieu Reymond, Valérie Méchin

**Affiliations:** 1 UMR 1318, Institut Jean-Pierre Bourgin, INRA, AgroParisTech, CNRS, Université Paris-Saclay, Versailles, France; 2 Weed Research Laboratory, Nanjing Agricultural University, China; 3 UR1268, Biopolymères, Interactions et Assemblages, INRA, Nantes, France; 4 Ecole Doctorale 567 Sciences du Vegetal, University Paris-Sud, University of Paris-Saclay, Orsay, France; University of Sao Paulo, BRAZIL

## Abstract

Plant cell walls development is an integrated process during which several components are deposited successively. In the cell walls in grass, the accessibility of structural polysaccharides is limited by the cell walls structure and composition mainly as a result of phenolic compounds. Here, we studied the patterns of cell walls establishment in the internode supporting the ear in three distinct maize genotypes. The developmental patterns observed in the internode cell walls in terms of its composition are reported with an emphasis on lignification, *p*-coumaroylation and feruloylation. We combined biochemical and histological approaches and revealed that internode cell walls development in maize before flowering is characterized by the rapid deposition of secondary cell walls components and robust lignification in both the pith and the rind. After flowering and until silage maturity, the slow deposition of secondary walls components occurs in the cortical region, and the deposited lignins are rich in β-O-4 bonds and are highly *p*-coumaroylated. We conclude the paper by proposing a revised spatiotemporal model based on that proposed by Terashima et al. (1993) for cell walls development in grass.

## Introduction

Grasses play a crucial role in animal nutrition for agriculture, both as fresh forage and as silage. Their valorization via the production of bioethanol is a current topic that has attracted intense research interest. In both cases, structural cell walls polysaccharides (cellulose and hemicelluloses) must be optimally valorized. The accessibility of structural cell wall polysaccharides is greatly limited by the cell wall structure and composition and especially by phenolic compounds such as lignins and *p*-hydroxycinnamic acids (ferulic and *p*-coumaric acids). Glucuronoarabinoxylan is the main hemicellulose encountered in grass cell walls. Nonxylan and noncellulosic polysaccharides only represent 10 to 15% of the cell walls [1]. An important feature in grass xylan compared to that in dicots is the presence of ferulic and *p*-coumaric acids in the arabinofuranosyl backbone. As reported in Hatfield et al. [1], ferulic acid content varies between grasses with genetic variability frequently shown in maize [2–7]. Hatfield and Chaptman [8] demonstrated the importance of the variability in ferulic acid content in different maize organs and tissues and especially its higher content in roots than in stems. In the stem, esterified ferulic monomers were slightly more abundant in the pith than in the rind. The role of ferulic acid in crosslinking has been studied extensively [6, 9–12]. Ferulic acid is effectively able to covalently link hemicelluloses and lignins. Ferulic esters thus serve as nucleation sites for lignification [5, 6], and one can predict the potential impact of ferulic ester availability in the cell walls on the lignification process. The ratio of *p*-coumaric acid to arabinoxylan in the cell walls is much lower and varies from undetectable to 1:15, which is 10- to 15-fold less than that of ferulic acid. The cross-linking of hemicelluloses or between hemicelluloses and lignins through *p*-coumaric acid has never been shown. In fact, *p*-coumaric acid is predominantly associated with lignins. Despite the very high levels of esterified *p*-coumaric acid (2 to 3-fold greater than esterified + etherified ferulic acids), its role in the grass cell walls is unclear. *p*-coumaric acid has been shown to be involved in the radical coupling of S units into the lignin polymer and is known to be ester-linked to the γ-position in the side chains of S lignin units [13]. Hatfield et al. have demonstrated that S units are enzymatically preacylated by *p*-coumaric acid in the cytosol [14]. These acylated monolignols are then incorporated into the lignin polymer by polymerization and copolymerization with traditional monolignols, resulting in acylated lignin [15]. In 2012, Withers et al. [16], characterized the first *p*-coumaroyl-CoA:monolignol transferase enzyme in rice. In 2014 a homologous enzyme was identified and characterized in maize [17]. Lignin is formed via the oxidative coupling of the three main monolignols, *p-*coumaryl alcohol, coniferyl alcohol, and sinapyl alcohol, which gives rise to *p*-hydroxyphenyl (H), guaiacyl (G), and syringyl (S) units interconnected by aryl ether bonds (β-O-4 linkages) and/or carbon-carbon bonds. The deposition of lignin in cell walls has far-reaching consequences for the mechanical stability, apoplastic solute conductance and pathogen resistance of forage crops [18, 19]. Meanwhile, the enzymatic degradability of forage declines during maturation because of the accumulation and progressive lignification of primary and secondary cell walls [20]. Until now, many studies of the genetics, genomics and biochemistry of lignin have been performed on grass and have revealed that the lignin content is the first factor that has been negatively correlated with cell wall degradability. However, a simple reduction in the lignin content may risk the loss of forage-enhancing agricultural fitness traits [21, 22]. Therefore, studies of the factors that contribute to cell wall degradability variations have focused on the influence of other cell walls parameters, such as lignin composition, structure and distribution and *p-*hydroxycinnamic acid content [3, 4, 7, 23–28]. Despite the abundance of data in the literature on cell walls biochemical factors and their impact on degradability, the role of these factors remains uncertain, and the conclusions that are reached are often contradictory. Several studies have suggested that cross-linking between lignin and polysaccharides through ferulic acid may be a major determinant of cell wall degradability; nevertheless, a consistent relationship has not always been observed for this parameter [3, 4, 29, 30]. Concerning lignin structure, it has been proposed that lignin rich in carbon-carbon bonds is more inhibitory in terms of cell wall degradability [20]. However, in a previously published study, we demonstrated that β-O-4-rich lignin has a more extended shape and is likely to adsorb along cellulose fibers, thus resulting in the decreased accessibility of polysaccharides for hydrolytic enzymes [3].

In stems, if several cell walls components influence cell wall degradability, the proportions of the different cell types could also strongly impact degradability [31, 32]. In 1991, Grabber et al. [33] showed that the sclerenchyma and the parenchyma were degraded by rumen fluid in stem cross-sections of switchgrass and orchardgrass after several hours. Jung and Casler [34] performed a similar study on maize stem cross-sections and showed that degradability differed between cell types. Méchin et al. [28] investigated the link between lignin distribution in maize stems, cell walls biochemical traits and cell wall degradability. These authors showed that highly digestible maize silage may be poorly lignified with lignin that is enriched in S units instead of G units and is preferentially localized in the cortical region instead of in the pith. An anatomical comparison of switchgrass stems selected because of their differences in degradability showed striking differences in the distribution of lignified cell types [35]. Overall, these works clearly indicated that differences in tissue lignification influence cell wall degradability at the organ level [36, 37].

Plant cell wall development is an integrated process during which several components are deposited successively. Some studies describe this sequential process in detail from a biochemical point of view [10, 38, 39]. The deposition model of cell wall polymers proposed in Terashima et al. in 1993 is still the essential reference [10]. Briefly, in this model, the authors proposed the successive formation of cell layers in the following order: middle lamella, primary walls, and outer, middle and inner layer of the secondary walls. They described the deposition of both carbohydrates and phenolic compounds. Concerning lignification, they argued that monolignols were produced sequentially from H, G and S lignin subunits and that lignins formed at an early stage were always more condensed than those formed at a late stage. They also stated that ferulic acids were incorporated earlier than *p*-coumaric acids in the cell walls. In contrast, to the best of our knowledge, there are no data concerning the influence of genetic variability in cell walls establishment in maize, except for a comparison between two hybrids that was performed by Boon et al. [39–41] via cell walls thickness and Van Soest measurements. In Jung [42] and in Jung and Casler [34], three maize hybrids were studied for two years throughout internode development; however, the data were averaged across the three genotypes for two years.

We present here a study that describes the patterns of cell walls establishment in three distinct genotypes selected for their similar lignin content and their differences in cell wall degradability at the silage stage. The study was conducted using the maize internode that supports the ear. The developmental patterns of the internode cell walls composition are reported with an emphasis on the patterns of lignification, *p*-coumaroylation and feruloylation. The objective was to identify whether genotypes with similarities in mature lignin content but with differences in cell wall degradability exhibited equivalent cell walls structure and reticulation and tissue lignification. The ultimate goal was to propose a developmental scheme for cell walls establishment.

## Materials and methods

### Plant material

Three inbred maize lines, F324, F66 and F7037, were grown at INRA Lusignan (Lusignan, 86, France) for three successive years (2009, 2010 and 2011). The trials used randomized block designs with three replicates. The plots consisted of one 5.2 m long row, and the density was 90 000 plants per hectare. Irrigation was applied from mid-June until the end of August to prevent water stress.

In 2009 and 2010, for the biochemical analysis, the principal ear internode was sampled at 6 stages of development beginning when the plants had reached the V8 stage (eight expanded leaves). The next five stages occurred when the plants underwent the V10 stage (ten expanded leaves), tasseling, and silking and at 14 days after the silking and silage stages. For the F66 genotype, V10 and tasseling stages were simultaneous. So, for this genotype only 5 stages were considered. For each inbred line, 50 internodes were sampled at the V8 and V10 stages, and 15 internodes were sampled at the four other stages. The internodes were chopped, oven-dried (60°C), ground with a hammer mill and passed through a 1 mm screen prior to the biochemical analyses.

During the summer of 2011, five ear internodes were sampled per developmental stage and were immediately placed in 70% ethanol:water (v/v) prior to subsequent histological analysis. The lengths and diameters (in the middle of the internode, across the short axes) of the internodes were measured with a ruler.

### Biochemical analyses

Cell wall residue was obtained from a two-stage extraction of the dry matter by ethanol and water [43]. The lignin content was estimated according to the Klason procedure [44] and expressed as the weight percentage of Klason lignin in the cell walls residue.

*The para*-hydroxycinnamic acid content was measured after treating the cell walls residue fractions with NaOH according to a previously described procedure [4], which involves two alkaline treatments with different levels of harshness. Esterified *p*-hydroxycinnamic acids were released by subjecting cell walls residue samples (50 mg) to a mild alkaline hydrolysis in 5 ml of 2N NaOH for 20 h at room temperature. Other cell walls residue samples (50 mg) were treated with 5 ml of 4 M NaOH for 2 h at 170°C to release both the esterified and the etherified hydroxycinnamates. The samples recovered from the mild and severe alkaline hydrolysis were subjected to the same precipitation procedure [45] prior to HPLC-MS analysis. The concentrations of the esterified *p*-coumaric and ferulic acids corresponded to the amounts of *p*-coumaric and ferulic acids released by the mild alkaline hydrolysis, whereas the concentration of the etherified ferulic acid was calculated as the difference between the amounts of ferulic acid released by the harsh and mild alkaline treatments [46].

The lignin monomeric composition was studied by thioacidolysis performed on 15 mg of cell walls residue placed in a screw-cap glass tube with 1 ml of internal standard (0.5 mg/ml each of C19, C21, and C22) and 10 mL of a dioxane/ethanethiol mixture (9:1, v/v) containing 0.2 M boron trifluoride etherate for 4 h in an oil bath at 100°C [47]. After the extraction of the lignin-derived monomers, the analysis of their trimethylsilyl derivatives was performed by GC/MS (Varian Saturn 2100, polydimethylsiloxane capillary column [SPB1, Supelco, 30m x 0.25 m, 0.25 μm], ion trap spectrometer detector [IE 70 eV, positive mode]). The molar yields of the H, G and S lignin-derived monomers was calculated on the basis of the Klason lignin content in the sample. The β-O-4 yield was calculated as the sum of the H, G and S molar yields.

We also estimated the percentage of S lignin units acylated by *p*-coumaric acid (S-PC) by assuming that all the *p*-coumaric acid was esterified into S lignin units [48]and extrapolating the percentage of S units measured in the uncondensed lignin (by thioacidolysis) to that in the whole lignin. Thus, S-PC was calculated using the following equation:

S‐PC=(100×esterifiedp‐coumaricacidcontent)/(Klasonlignincontent×%S).

All biochemical analyses were performed in duplicate.

### Anatomical analyses

A 1-cm-long segment was sampled from the middle of each of the five internodes and soaked in water overnight. For each segment, 15 150-μm thick serial stem cross-sections were prepared with a vibratome (HM 650 V Vibratome from MicroMicrotech France).

The slices were imaged using the “BlueBox” macroimaging system developed at INRA Nantes [49]. An optical fiber ring (SCHOTT DCRIV Light Source, Mainz, Germany) provided controlled darkfield illumination. Mosaic images were acquired that were approximately 4500 x 4500 pixels in size with a pixel size of 3.6 μm x 3.6 μm. The grey values were coded within a range from 0 (black) to 255 (white). As the diameters of most parenchyma cells ranged between 50 and 150 μm, the resolution of the macroscopy images made it possible to observe the cellular morphology and the whole slices simultaneously.

Images were analyzed by using grey level granulometry, which involves applying image transformations to the structuring elements of increasing size [50, 51]. The method can be compared to the sieving of the images and the structuring elements to the mesh of the sieve. By applying morphological filters of increasing size and a square as a structuring element, cells are progressively removed from the tissue images. A granulometric curve is built by measuring the variations in the sums of the grey levels after each closing step. The granulometric curves can be compared to typical granulometric distributions except that they are calculated by taking grey level variations into consideration. The geometrical mean of the distribution was used as a summary parameter that described the average size of the cells in a given image [51].

### Histological analyses

The stem cross-sections were stained overnight using a Fasga solution diluted in distilled water (1:8, v/v). The Fasga solution was composed of 0.05% safranin O (SIGMA S8884), 0.2% Alcian blue (SIGMA S2889), 1.5% acetic acid and 46% glycerin (SIGMA G7893) in distilled water. Safranin is a red basic cationic dye, and Alcian blue is an acidic anionic dye. The Fasga solution stained the lignified tissues red, whereas the nonlignified or poorly lignified tissues were stained blue. After staining, the sections were rinsed twice for 5 min with distilled water. The sections were examined under a magnifying glass (×1, Nikon SMZ 1000) and were digitalized as color images by a ProgRes C3 digital microscope camera with a resolution of 10 μm per pixel. Color was represented by the three red, green, and blue channels, each of them being coded between 0 and 255.

Each image was automatically numerically analyzed in ImageJ freeware with a plugin that is described in Zhang et al. [52] to segment and evaluate the red/blue intensity ratio relative to the distance to the outer epidermis for the six stages of development. On each image, morphological image filtering was applied to preserve the local color of the tissue. The region corresponding to the stem section was identified and divided into a fixed number of concentric regions of interest. The average color within each region of interest was measured, resulting in curves that described relationship between the variations in tissue color and the distance to the epidermis. Afterward, the change in the lignification rate in each region (pith, rind and blue ring) in the stem sections during internode development was assessed. Finally, we measured the average intensity of the red, blue and green areas in the whole cross-sections for each genotype and each stage to establish the average profile for each RGB channel during development for each of the three genotypes.

### Statistical analysis

Any block effect or the interaction of any other factor with the block effect was measured. Thus to compare the relative influences of genotype, year and stage the variance analysis was carried out using R 3.0 (CRAN-project, lm function) according to the following model:

Vijkl=μ+Yi+Gj+Sk+YixGj+YixSk+GjxSk+Rijkl

where Vijkl is the value of genotype j, in year i, for stage k and for block l; μ is the overall mean; Yi denotes the main effect of year i; Gj denotes the effect of genotype j; Sk denotes the main effect of stage k; Yi x Gj denotes the interaction effect between genotype j and year i; Yi x Sk denotes the main effect of stage k nested in year i; Gj x Sk denotes the main effect of genotype j nested in stage k; Rijk is the random residual term.

The data were combined in terms of year, field and analysis replicates before the determination of the correlation coefficients using R (cor function).

## Results and discussion

Three public early/medium-early inbred maize lines were selected on the basis of results obtained from experiments performed in 2006 and 2008 at INRA Lusignan [53]. F324, F66 and F7037 lines were chosen because they exhibit comparable lignin contents (5.2, 4.8 and 5.1% of Van Soest lignin (ADL) in the cell walls, respectively) and significantly different cell wall degradabilities (38.8, 34.8 and 27.6% of the cell walls, respectively) at the silage stage. Cell walls deposition was studied throughout plant development in these three inbred lines to elucidate the applicability of one or more cell walls developmental models both biochemically and in terms of lignin distribution within the different stem tissues.

### Influence of genetic variation on internode elongation, cross-section surface and cell size in the three genotypes throughout internode development

**Fig 1:**
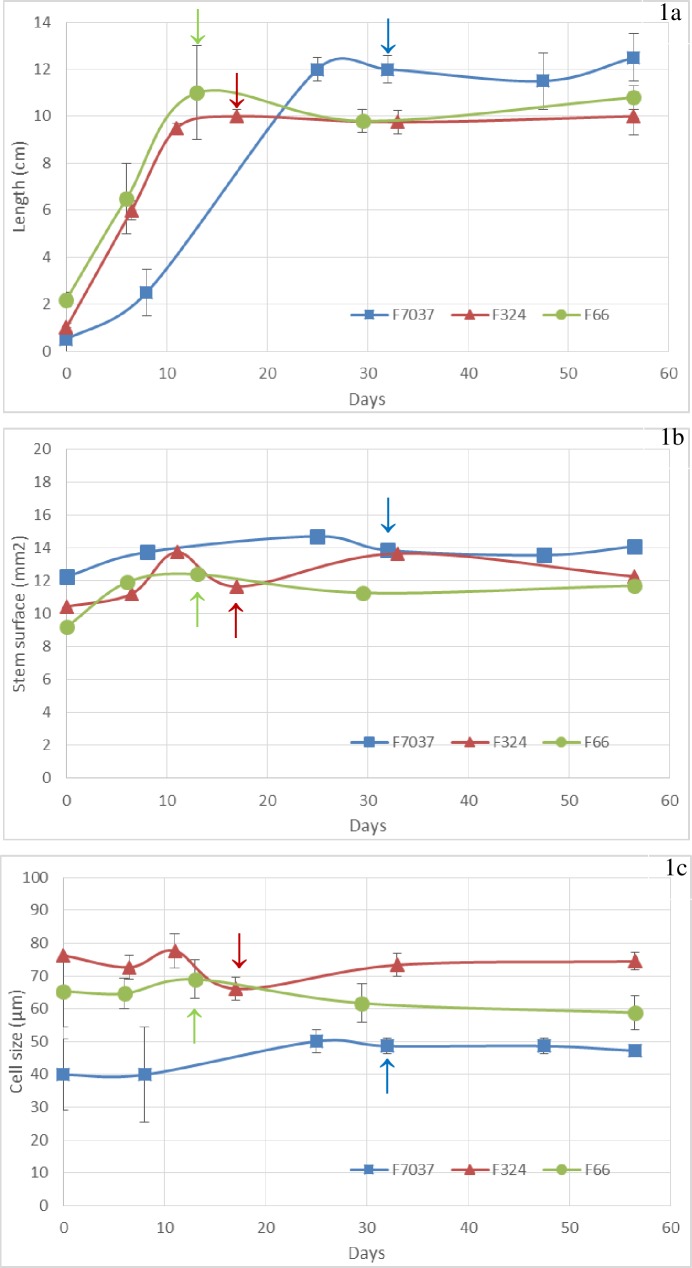
Changes in the morphological parameters during internode development in the three genotypes. Fig 1A: length of the principal ear internode, Fig 1B: cross-section surface and Fig 1C: cell size of the parenchyma cells. Data were averaged across the three blocks collected in 2011. Arrows = silking date. Smoothed curves were used only for graphical purposes.

The length of the principal ear internode was measured at each developmental stage for each genotype. The three inbred lines exhibited different elongation patterns during development (S1 Table and Fig 1A). F66 and F324 were the two early genotypes and exhibited rapid growth compared to F7037, which was medium-early and exhibited slower elongation. Ten centimeters in length was reached in approximately 10 days for F66 and F324 and after more than 20 days for F7037. However, it should be noted that all 3 genotypes completed their elongation at or just before silking, regardless of the silking date. This physiological stage marked the end of internode elongation in all genotypes that were examined. At silage maturity, the length of the internode in F7037 was greater than that in the other two lines. Scobbie et al. [54] showed that when elongation ceased in maize internode, cells near the upper part of the internode had already begun to undergo extensive secondary walls deposition, whereas at the bottom of the internode, the cell walls appeared to still be primary in nature. Similarly, Jung [42] indicated that cell walls development shifted from a combination of primary and secondary walls deposition to only secondary walls development after the end of the elongation of the internode. Thus, in our three inbred lines, the shift in the developmental pattern of internode elongation should be associated with different processes during cell walls development/deposition over time. These differences should be visible in our in-depth biochemical and histological characterizations.

The change in the internode diameter was less important than that in the length and was also complete when elongation ended in the three inbred lines. At the silage stage, F7037 showed a larger internode diameter (1.33 ± 0.01 cm) than F324 (1.20 ± 0.05 cm) and F66 (1.13 ± 0.02 cm). The stem cross section surface did not change considerably after the second stage (V10, Fig 1B). The average cell size (Fig 1C) was stable within each line over time but was different between lines. Indeed, a twofold variation was observed between the F7037 line, which exhibited a 45 μm cell size, and the F324 line, which exhibited a 75 μm cell size. Thus, F7037 had the largest internode diameter but exhibited the smallest cell size, which implied that it had an increased cell walls density compared to that of the other two genotypes that was potentially associated with its lower degradability.

### The primary differences in internode elongation and cell walls phenolic composition were observed in early stages before silking

The variance analysis of the cell walls biochemical composition indicated that throughout internode development, the three inbred lines differed for most biochemical parameters. Indeed, the genotype effect was significant (P < 0.001) for the Klason lignin content, G subunits, β-O-4 yield, *p*-coumarate esters and ferulate esters (Table 1). In previous studies, no differences in the pattern throughout development have been noted [34, 42, 55]. The inbred lines used in our study were chosen to limit the variation in lignin content in the cell walls at the silage stage. They showed significant differences in phenolic composition during internode development, suggesting that the walls pattern of lignification in the cell walls differed. This is the first time, to our knowledge, that the influence of genetic variation on cell walls phenolic composition during internode development has been reported.

**Table 1:** ANOVA results of the fixed effects on the walls biochemical composition of cell walls in the principal ear internode for the three maize lines (F324, F66, and F7037) grown in 2009 and 2010 and sampled during 6 developmental stages.

		Variance effects					
		Y_i_	G_j_	S_k_	YG_ij_	YS_ik_	GS_jk_
All stages	L Internode	ns	*	***	ns	ns	**
	KL	***	***	***	ns	***	***
	G	***	***	***	**	***	ns
	S	***	**	***	ns	***	***
	β-O-4 yield	***	***	***	ns	***	**
	S/G	ns	ns	***	***	ns	***
	PC est	***	***	***	ns	***	***
	SPC	ns	**	***	ns	*	***
	FA est	***	***	***	*	***	***
	FA eth	***	ns	***	ns	*	***
V8 to silking stage	L Internode	ns	ns	***	ns	ns	**
	KL	***	***	***	ns	**	***
	G	***	***	***	ns	***	ns
	S	***	*	***	ns	***	**
	β-O-4 yield	***	***	***	ns	***	*
	S/G	ns	ns	***	*	*	***
	PC est	***	***	***	**	***	***
	SPC	ns	ns	***	ns	*	**
	FA est	***	***	***	ns	***	***
	FA eth	ns	**	***	ns	ns	***
Silking to silage stage	L Internode	ns	***	ns	ns	ns	ns
	KL	*	***	*	*	ns	ns
	G	***	**	**	**	ns	ns
	S	***	***	***	ns	ns	ns
	β-O-4 yield	***	***	***	*	ns	ns
	S/G	ns	ns	***	**	ns	ns
	PC est	ns	***	***	ns	ns	ns
	SPC	ns	***	**	ns	*	*
	FA est	***	***	***	ns	ns	**
	FA eth	***	ns	*	ns	ns	ns

*: P < 0.05

**: P < 0.01

***: P < 0.001

ns: P > 0.05.

L internode: length of the internode supporting the ear; KL: Klason lignin content in the cell walls; G and S: molar yield of β-O-4-linked G and S lignin-derived monomers calculated on the basis of the Klason lignin content in the cell walls; β-O-4 yield: sum of the yields of the H, G and S β-O-4-linked lignin-derived monomers; S/G: S/G subunit ratio; PC est: esterified *p*-coumaric acid content in the cell walls; SPC: percentage of S lignin units acylated by p-coumaric acid; FA est: esterified ferulic acid content in the cell walls; FA eth: etherified ferulic acid content in the cell walls.

As observed in previous studies [34, 42], the stage effects were significant (P < 0.001) for all cell walls phenolic parameters. To further investigate the stage effects, variance analyses were conducted for two distinct periods: from V8 to silking and from silking to silage (Table 1). The variance analysis carried out for each period indicated no significant genotype x stage interaction occurred in the late stages (Table 1). Thus, in the late stages, the accumulation of cell walls components in the three inbred lines followed a similar pattern. The main differences in all phenolic parameters thus occurred in an earlier stage before silking.

### Different developmental patterns were observed for lignin content, composition, structure and distribution

#### Changes in lignin content throughout internode development

**Fig 2:**
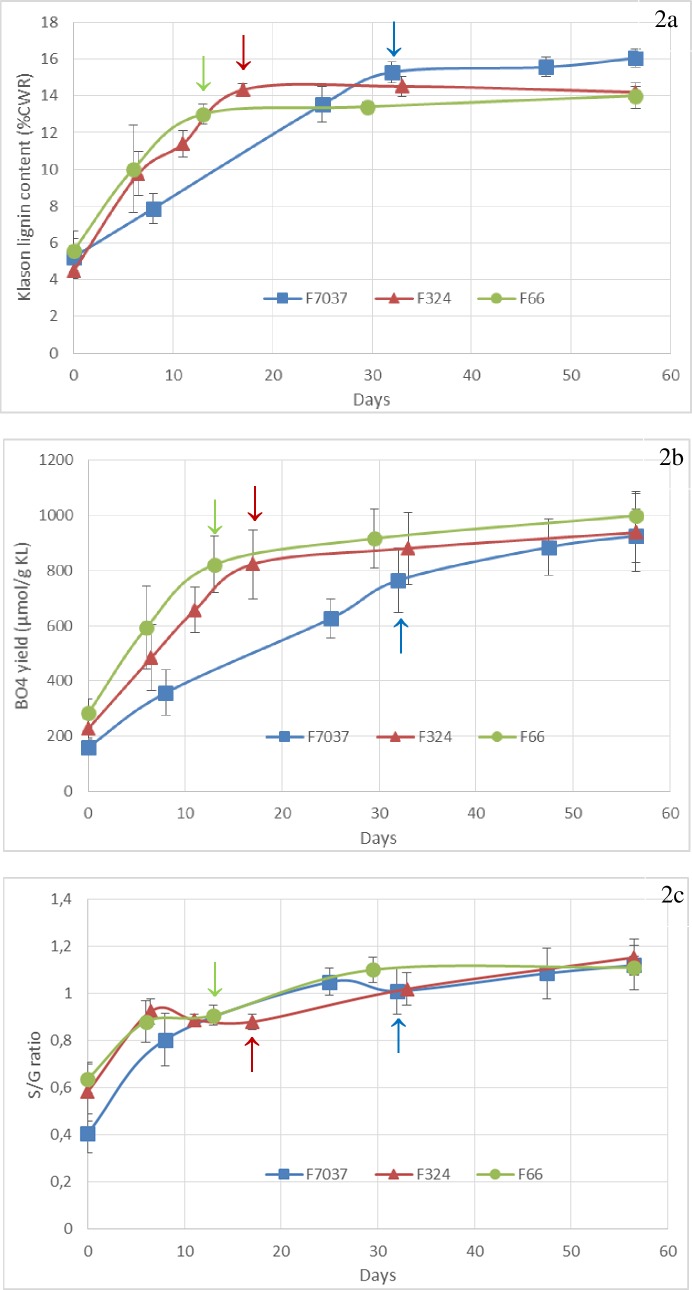
Changes in the lignin content, composition and structure during internode development in the three genotypes. Fig 2A: Klason lignin content, Fig 2B: lignin units involved only in β-O-4 bonds (uncondensed lignin) and Fig 2C: S/G thioacidolysis ratio. Data were averaged across the blocks collected in 2009 and 2010. Arrows = silking date. Smoothed curves were used only for graphical purposes.

The three genotypes were initially chosen because they exhibit similar ADL lignin content in the cell walls at the silage stage (between 4.8 and 5.2%). The Klason lignin content in the cell walls in each of these three genotypes at the silage stage was ultimately similar (14% for F66 and F324 and 16% for F7037, Fig 2A). As mentioned in Hatfield and Fukushima [56], when applied to grasses, the acid detergent solution used in the Van Soest procedure dissolves more than 50% of the lignin, which explains the dramatically lower values obtained for the ADL lignin content compared to the values obtained with the Klason procedure. As detailed in Zhang et al. [3], the lignin contents estimated by the Van Soest (ADL) and the Klason methods differ because the methods measure different types of lignin. The Klason lignin value is a better estimation of the whole lignin content in the cell walls, whereas the ADL lignin value represents mainly the condensed (C-C linkages) lignin content.

Among the three genotypes, Klason lignin content was similar in the first stages of development (4.5 ± 0.5%, 5.6 ± 1.0% and 5.2 ± 1.0% for F324, F66 and F7037, respectively, S1 Table and Fig 2A). A rapid increase in Klason lignin content was observed for the three genotypes in the first developmental period between the V8 and Silking stages (Fig 2A). At the silking stage, the Klason lignin content reached its peak in all three genotypes (Fig 2A). F66 and F324 thus accumulated approximately 10% of the lignin in their cell walls in only 2 weeks, while F7037 accumulated approximately 10% of the lignin in its cells walls after more than 4 weeks (Fig 2A). After silking, in the internode supporting the ear, the lignin content increased negligibly for all three genotypes (Table 1 and Fig 2A).

#### Changes in lignin structure and composition throughout development

The proportions of the H, G and S lignin subunits was evaluated by thioacidolysis and thus reflect the lignin units involved only in β-O-4 bonds. The total β-O-4 yield provides an estimate of the proportion of lignin units involved only in β-O-4 bonds (β-O-4 yield), which represent the so-called “uncondensed” portion of the lignin polymer. An important effect of stage (P < 0.001, Table 1) was observed for the β-O-4 yield; a consistent increase was observed in the uncondensed portion of the lignin polymer in the principal ear internode during internode development (S1 Table and Fig 2B).

At earlier stages, the three studied genotypes exhibited lignin with a poor proportion of β-O-4 linkages. As described by Terashima et al. [10], lignin formed at an early stage was always more condensed than that formed at a late stage. The three genotypes did not differ in their developmental pattern in terms of β-O-4 yield but differed in their developmental pattern in terms of Klason lignin content. Between the silking and silage stages, approximately 150 μmol.g^-1^ Klason lignin consisting of β-O-4 lignin units was deposited, while the total lignin content increased only slightly. Therefore, β-O-4 was probably the main linkage formed during this late, slow lignification period. Rapid polymerization favors the C-C coupling of monolignols; in contrast, gradual polymerization favors β-O-4 coupling between monolignols to form lignin polymers [57]. This is in accordance with our results, which showed that rapid lignin accumulation was certainly the place for mixed bonding pattern within the lignin polymer that was characterized by C-C bonds, whereas the slow and late lignin accumulation period resulted in a β-O-4 bonding pattern within the lignin polymer.

We can speculate on whether the establishment of β-O-4 bonds is only constrained by chemical features (oxidative coupling, reactivity, quinone stability, etc.) or if genetic determinism is involved in the spatiotemporal regulation of this pattern; however, the debate of this question is beyond the scope of this paper.

In the first stage of development, the relatively low syringyl to guaiacyl ratio (S/G ratio) was consistent with the well-known shift in H, G and S lignin subunit deposition during lignification, as described by Terashima et al. [10]. We observed that G unit accumulation ceased before S unit accumulation, which continued until the last stage (S/G unit > 1, Fig 2C). Surprisingly, the S/G unit ratio change for F7037 was quite synchronous with that of F66 and F324 despite the crucial delay in their lignin deposition. Thus, during more than half of the accumulation phase, F7037 accumulated more S units than G units, in contrast to the other two genotypes (Fig 2C). Therefore, in addition to the lignification model for Gramineae proposed by Terashima et al. [10], we believe that other lignification models may be plausible. High genetic variability is present in maize (and more generally in grasses), which influences the timing of lignin composition changes even if the S/G unit ratio increases throughout development. Jung and Casler [34] stated that the shift from guaiacyl-rich lignin to syringyl-rich lignin is a general phenomenon of secondary cell walls development in grass tissues, and Terashima et al. [10] proposed that when lignification reached the secondary cell walls, the syringyl-rich lignin content and the S/G unit ratio increased. This would indicate that, for F7037, either the deposition of the secondary walls started earlier or the primary walls was richer in S units.

#### Changes in lignin distribution at the tissue level throughout internode development

**Fig 3:**
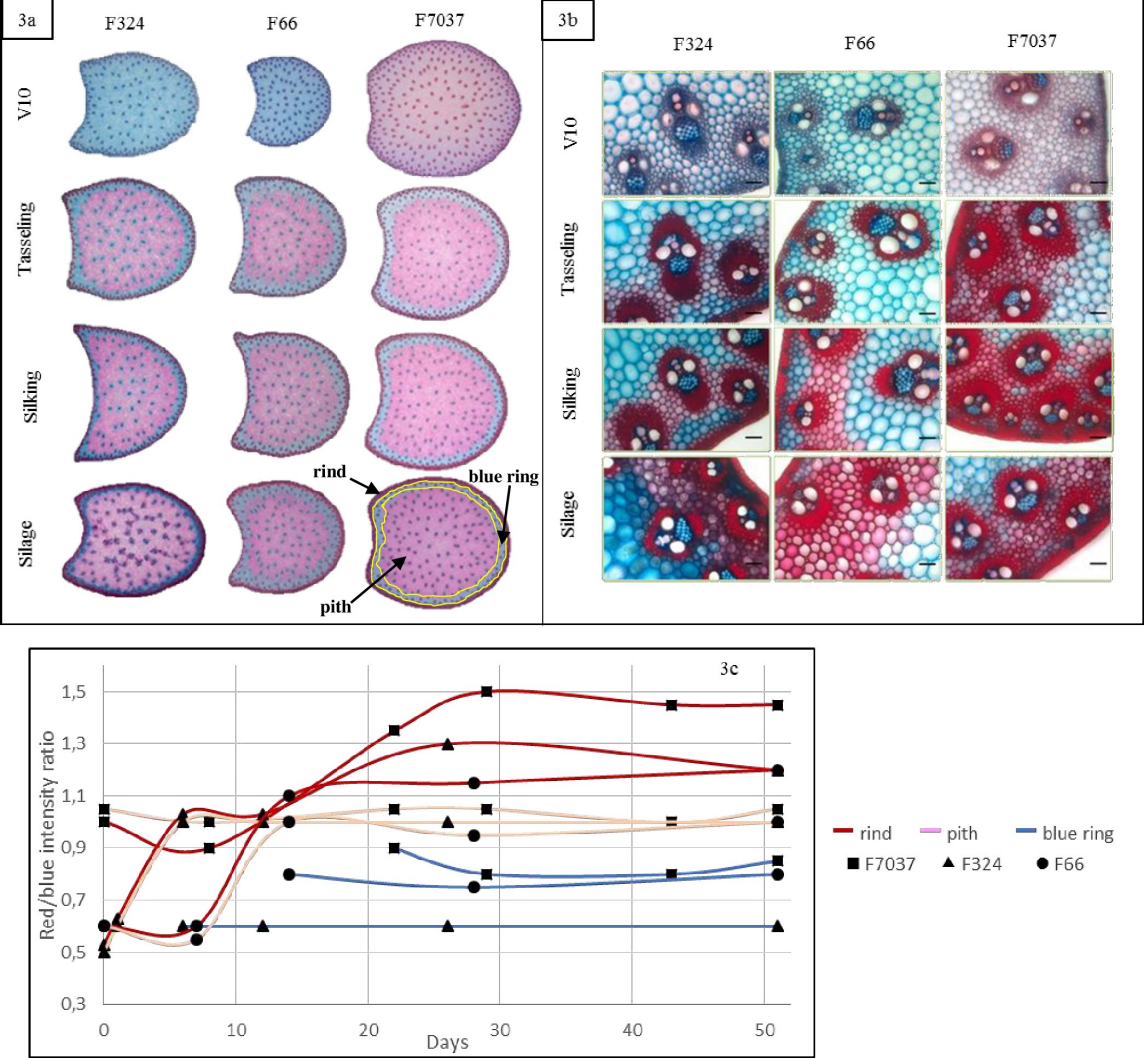
Changes in tissue lignification during internode development in the three genotypes. Fig 3A: whole internode Fasga-stained cross section. Pith, blue ring and rind were indicated on the F7037 cross section at silage stage, Fig 3B: rind region of the Fasga-stained cross section (bar = 100 μm) and Fig 3C: red/blue intensity ratio for each tissue (red = rind, blue = blue ring and pink = pith). Smoothed curves were used only for graphical purposes.

Tissue lignification during internode development was observed via Fasga staining. The lignified tissues were stained red, whereas the non-lignified or poorly lignified tissues were stained cyan/blue. For each Fasga-stained cross section from the three genotypes during the 6 developmental stages (Fig 3A), we evaluated the red/blue intensity ratio relative to the distance to the outer epidermis using a software plugin developed and described in Zhang et al. [52]. We defined three main regions on the basis of the color profiles that corresponded to the pith (central zone indicated on the F7037 cross section at silage stage, Fig 3A), the blue layer in the pith (median zone indicated on the 7037 cross section at silage stage, Fig 3A) and the rind (external zone indicated on the F7037 cross section at silage stage, Fig 3A. We assessed the changes in the lignification rate in each region at each stage for each genotype (Fig 3C).

The blue ring appeared in the third stage (tasseling) for all 3 genotypes. The lignification of this poorly lignified parenchymal region was minimal and did not change during internode development after the tasseling stage.

As presented in detail for F324 in Zhang et al. [52], a great increase in the red/blue intensity ratio in the rind and pith regions in all 3 genotypes between V10 (stage 2) and tasseling (stage 3) was associated with the lignification of these two regions. In the rind region, the relatively high values of the red/blue intensity ratio indicated that the cells in this region were much more lignified than those in other regions. After tasseling, the red/blue intensity ratio in the pith remained steady, whereas in the rind, this ratio continued to increase (Fig 3C). Thus, lignification in both the blue ring and pith regions stopped after tasseling. Therefore, the lignins that accumulated after this stage were deposited predominantly in the rind region. This is clearly illustrated in Fig 3A and in Fig 3B, which shows the intense lignification in the rind region in all 3 genotypes after tasseling.

Upon comparison of the 3 genotypes, the three main observations are that i) the highest blue staining intensity was found in the blue ring in F324, ii) the highest red/blue intensity ratio was found in the rind in F7037 when compared to F66 and F324 and iii) the profiles of lignification in the pith region were comparable for the 3 genotypes. F7037 was thus a more intensely lignified genotype overall and exhibited a highly lignified rind.

In conclusion, lignification occurs largely before the flowering stage both in the pith and in the rind. After flowering and until maturity, the deposited lignins are very rich in β-O-4 bonds and are mainly localized in the rind.

### Throughout internode growth, different developmental patterns were observed in terms of p-hydroxycinnamic acid accumulation

#### Changes in esterified p-coumaric acid content throughout internode development

**Fig 4:**
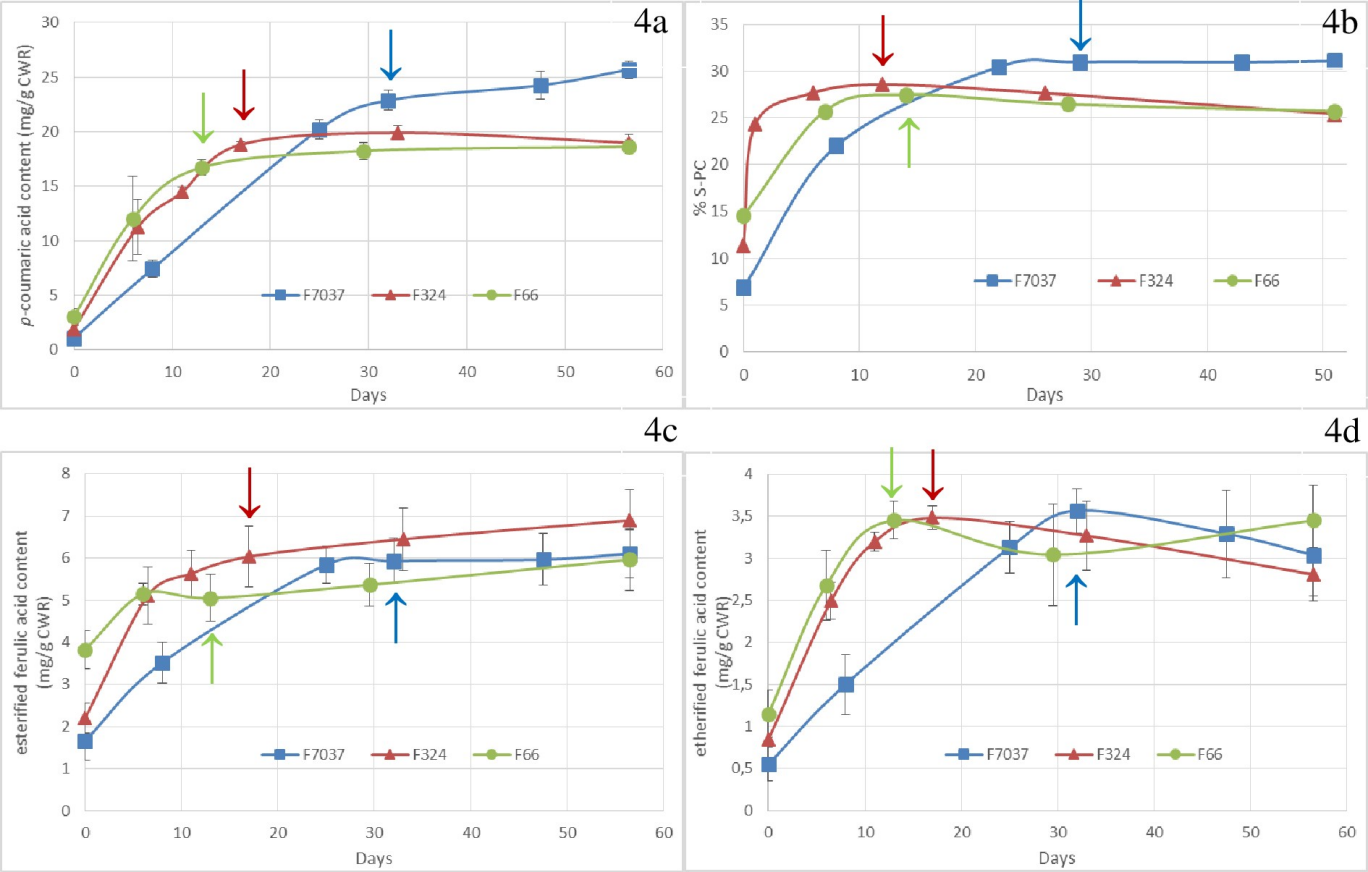
Changes in p-coumaroylation and feruloylation content during internode development in the three genotypes. Fig 4A: esterified p-coumaric acid content, Fig 4B: percentage of S lignin subunits acylated by p-coumaric acid, Fig 4C: esterified ferulic acid content and Fig 4D: etherified ferulic acid content. Data were averaged across the blocks collected in 2009 and 2010. Arrows = silking date. Smoothed curves were used only for graphical purposes.

The deposition of *p*-coumaric acid followed the same pattern as that observed for the Klason lignin content (S1 Table and Fig 4A). A strong correlation was observed between esterified *p*-coumaric acid content and Klason lignin content in both the early (R^2^ = 0.98 from stage 1 to 4) and mature stages (R^2^ = 0.94 from stage 5 to 6).

The %S-PC increased dramatically in the earlier stages and nearly reached its maximum in V10 (stage 2) for F66 and F324 by increasing from 12–15% to 25% in just a few days. However, for F7037, the %S-PC increased from 7% to 30% in 3 weeks. Analysis of variance showed a significant genotype effect for *p*-coumaric acid content and %S-PC in the mature stages. At the silage stage, large genotypic variations were observed in terms of *p*-coumaric acid content and %S-PC. F7037, which had the highest Klason lignin content, also had the highest *p*-coumaric acid content and %S-PC value. F324 and F66 had similar Klason lignin content and presented similar *p*-coumaric acid content and %S-PC values.

In grass, *p*-coumaric acid is mainly esterified via the γ-position in the side chains of S lignin units, which allows *p*-coumaric acid accumulation to serve as an indicator of lignin deposition [58]. Several studies have shown a positive relationship between the *p*-coumaric acid content in the cell walls and the level of lignin [8, 46, 54]. The current study reinforces this observation. During maize cell walls development, the esterified *p*-coumaric acid content increased according to the same trajectory followed by the Klason lignin content.

It is well established that the *p*-coumaric acid content in the cell walls dramatically increases during secondary cell walls deposition in maize and that *p*-coumaric acid is mainly associated with syringyl-rich lignin [34]. Recent work has demonstrated that S units are enzymatically preacylated by *p*-coumaric acid in the cytosol [14]. These acylated monolignols are then incorporated into the lignin polymer by polymerization and copolymerization with traditional monolignols, resulting in acylated lignin [15]. The role of lignin acylated by *p*-coumaric acid is still unclear. It is clear that the *p*-coumaroylated S unit is crucial for radical transfer mechanisms that improve the incorporation of S units into lignin polymers. After stage three, the %S-PC value leveled off. This plateau was higher for F7037 than for F324 and F66. Thus, approximately 31% of the S units appeared to be acylated by *p*-coumaric acid in F7037, whereas approximately 25% were acylated in the two other genotypes. We have shown that in the late stages, uncondensed β-O-4 lignin was mainly formed; this lignin was thus also strongly p-coumaroylated. The enzymatic step of S unit acylation by *p*-coumaric acid could thus be a key step in controlling the final structure and composition of lignin. This is especially apparent when considering the results obtained and described in detail previously in Zhang et al. [3], which demonstrated that *p*-coumaroylated S lignin units were a major limiting factor for cell wall degradability when lignin content variation was small. Works by Withers et al. [16] on an acyltransferase that catalyzes the acylation of monolignols in grass suggest the possibility of modifying gene expression and thus modulating and regulating acylation in plants. In a recent study, Sibout et al. [59] introduced a Brachypodium *p*-coumaroyl-coenzyme A monolignol transferase in Arabidopsis and succeeded in altering the Arabidopsis lignin content, structure and composition and consequently impacting cell wall degradability.

#### Esterified ferulic acids were deposited throughout plant development but anchored lignification only in the early stages

Variance analysis indicated that the three genotypes differed in their esterified ferulic acid content (FAest) throughout development (P < 0.01) (Table 1). In grass, ferulic acids are esters linked to arabinoxylans and deposited in the primary cell walls [60]. Once they are incorporated into the cell walls, peroxidases oxidize ferulic acid to form several possible ferulic acid dimers [61] and also couple ferulic acid to monolignols [62]. Ralph et al. [6] suggested that esterified ferulic acid probably serves as an initialization site for lignification. In the present study, the level of esterified ferulic acid was relatively high in the first stage, especially for F66 (S1 Table and Fig 4C). This early deposition of esterified ferulic acid should play a crucial role in the development of lignification in the primary cell walls. Esterified ferulic acid accumulation was high during the first three stages and slowed down once secondary walls deposition began. Esterified ferulic acid accumulation continued during the late stages for F66 and F324, but surprisingly did not continue for F7037 (Fig 4C). Changes in ester-linked ferulic acid are always somewhat difficult to interpret mostly in late developmental stages where cell wall complexity limit released by mild alkali. Our results are yet consistent with those presented by Jung (2003) that indicate that esterified ferulic acid deposition is not limited to primary cell walls but that approximately 60% of esterified ferulic acid is present when secondary walls formation begins in maize. In 2008, Hatfield et al. [63] reported a constant level of ester-linked ferulic acid in internode tissues in developing maize stems, thus demonstrating that ferulic acid was continually incorporated into secondary cell walls as well as primary walls. The highest amount of etherified ferulic acid was observed in the third stage. Etherified ferulic acids are proposed to link hemicelluloses and lignins. After stage three, the etherified ferulic acid content did not increase further, suggesting that no supplementary links between these two polymers was established (Fig 4D). The possibility remained that feruloyl arabinoxylan polymers continued to be laid down in the secondary walls, but no more ferulic acid radically couple them to monolignol. This is consistent with the fact that lignin synthesized during the last stages was mainly composed of β-O-4 bond subunits. The predominance of β-O-4 bonds was an indicator of an endwise mechanism similar to that described by in Demont-Caulet et al. [64]. Thus, at the end of plant development, few lignins were synthesized, but the new lignins were not anchored to new ferulic acid primers but were elongated via β-O-4 bonding of the existing lignins. Ferulic acid primers were thus recruited before stage 4 to anchor lignification.

#### Synthesis of the biochemical and histological findings to propose a spatiotemporal model of cell walls development

**Fig 5:**
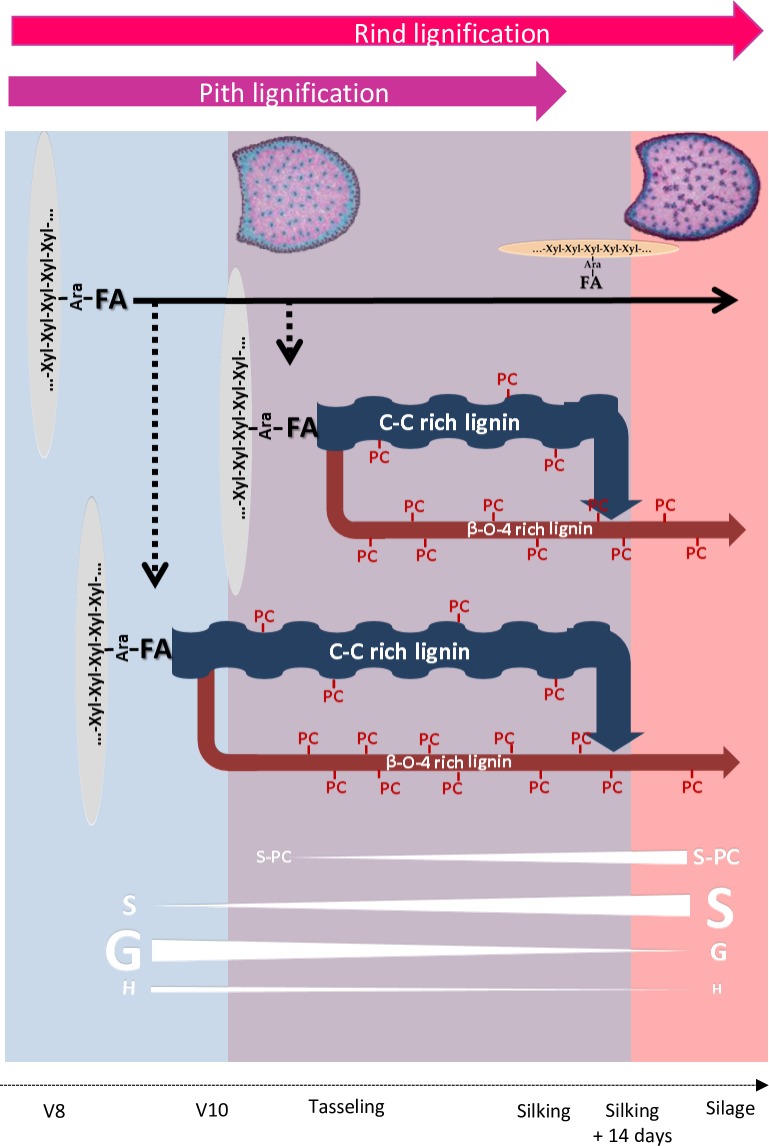
Schematic illustration of the spatiotemporal evolution of the internode cell walls in maize. Blue part of the figure symbolize the period of fast deposition of the primary cell walls. Purple part of the figure symbolize the period of fast deposition of the secondary lignified cell walls. Pink part of the figure symbolize the period of slow deposition of the secondary cell walls in the rind region of the maize internode at late stages. Pith lignification (purple arrow) and Rind lignification (red arrow) both take place until silking. After silking lignification only occurred in rind. β-O-4 rich lignin: β-O-4 bonds between lignin subunits were established during all the cell wall internode lignification. From silking to silage few lignins were synthesized but these lignins are very rich in β-O-4 bonds. These lignins deposited in late stages were mainly present in the rind region. C-C rich lignin: lignin with less β-O-4 bonds. Xyl-Xyl-Xyl: xylose backbone of the main maize hemicelluloses. Ara: main maize hemicelluloses were arabinoxylanes. Ferulic (FA) was ester linked to Ara. These ferulic primers accumulated during all internode development even if new ferulic primers were not recruited for lignification after silking. PC: p-coumaric acid was mainly link to S lignin units. PC accumulated until the end of internode lignification. Thus, highly linear p-coumaroylated lignin accumulated in rind region at the end of lignification from silking to silage stage.

In the present study, we combined biochemical and histological approaches to characterize cell walls deposition and lignification during maize stem development. Internode cell walls development in maize occurred during three different steps: (1) fast deposition of the primary cell walls in whole cross sections; (2) fast deposition of the secondary cell walls; (3) slow deposition of the secondary cell walls in the cortical region (Fig 5).

The spatial and temporal deposition of cell walls components and the development of the cell walls structure varied during these three cell walls developmental steps and were also clearly genotype dependent. In the primary cell walls, esterified ferulic acid was predominantly deposited, and the large influence of genetic variation on the esterified ferulic acid content was likely to be critical for the variation of lignification in the primary cell walls. In the second step, the fast deposition of lignins with both C-C and β-O-4 bonds led to the fast deposition and lignification of the secondary cell walls. Once the fast deposition of lignin with C-C bonds ended, lignins with β-O-4 bonds were likely the primary lignins formed during the late stages of lignification and were predominantly deposited in the cortical region. This cortical region thus appeared to be very specific in terms of its composition. Our results suggested that the cortical region was highly lignified with lignin rich in β-O-4 bonds and highly p-coumaroylated. Barros-Rios et al. [31] compared the cell walls composition in separated maize pith and rind tissues. They demonstrated that rind tissues were less degradable than pith tissues and richer in etherified ferulic acids, esterified p-coumaric acids and were more lignified. Similarly, Hatfield and Chapman [8] demonstrated that pith tissues had lower levels of lignin and *p*-coumaric acid. As established in Zhang et al. [3] highly *p*-coumaroylated lignin rich in β-O-4 bonds reduced cell wall degradability. Thus, the spatial and temporal developmental scheme of the cortical region made it a highly undegradable and resistant tissue. This reinforced our recent results described in detail previously [7] and suggesting that drought, induce a decrease of internode lignification associated with a preferential lignification and *p*-coumaroylation of the rind region to maintain plant posture and resistance. In line with the results described by Hatfield and Chapman [8], we propose that *p*-coumaric acid has an active role in the formation of lignin in grass cell walls. In addition, the results presented in this publication also underline the fact that the distribution of lignin at the tissue level is important to take into account to better understand the influence of genetic variation on overall cell walls composition and the degradability of internode tissues. In our opinion, the impact of the distribution of lignified tissue on cell wall degradability is critical and has long been underestimated.

The selection of lines with improved cell wall degradability should consider the distribution of lignin and *p*-hydroxycinnamic acid in tissue in terms of both quantity and composition.

## Supporting information

S1 Table Data set. Morphological parameters, biochemical composition of cell walls and red/blue intensity ratio for each tissue of the principal ear internode of three maize lines (F324, F66, and F7037) sampled at 6 developmental stages.
(XLSX)

## Abbreviations:

G: guaiacyl lignin unit
H: *p*-hydroxyphenyl lignin unit
S: syringyl lignin unit
V8 stage: eight expanded leaves stage
V10 stage: ten expanded leaves stage
